# Conservative Management of Medication-Related Osteonecrosis of the Jaws (MRONJ): A Retrospective Cohort Study

**DOI:** 10.3390/antibiotics10020195

**Published:** 2021-02-17

**Authors:** Elena M. Varoni, Niccolò Lombardi, Giulio Villa, Alberto Pispero, Andrea Sardella, Giovanni Lodi

**Affiliations:** Dipartimento di Scienze Biomediche, Chirurgiche ed Odontoiatriche, Università degli Studi di Milano, Via Beldiletto 1, 20142 Milan, Italy; niccolo.lombardi@unimi.it (N.L.); giulio@tuilik.com (G.V.); pispero.alberto@gmail.com (A.P.); andrea.sardella@unimi.it (A.S.); giovanni.lodi@unimi.it (G.L.)

**Keywords:** antibiotics, pentoxifylline, tocopherol, sequestrectomy, osteonecrosis

## Abstract

Background: Medication-related osteonecrosis of the jaw (MRONJ) is a severe side effect of bisphosphonates and anti-resorptive drugs prescribed for treatment of severe osteoporosis, Paget’s disease, and bone malignancies. The aim of this study was to evaluate the clinical outcome of a combined pharmacological and surgical management strategy on patients affected by MRONJ. Materials and methods: Medical records of patients with MRONJ were retrospectively examined to collect clinical history data. Conservative management included an initial pharmacological phase with antibiotics and antiseptic agents, followed by surgical intervention to remove bone sequestrum. Primary outcomes were healing from MRONJ at short term (1 month after surgery) and at longer term (3 months after surgery). Secondary outcome was assessment of recurrences at longer-term follow-up. Results: Thirty-five patients were included in the study with mean follow-up of 23.86 ± 18.14 months. Seven cases showed spontaneous exfoliation of necrotic bone during pharmacological therapy, which in one case did not require any further intervention. At 1-month posttreatment, 31 out of 35 (88.5%) patients showed complete healing. The 25 patients who were followed for at least 3 months revealed a healing rate of 92% (23/25). Recurrences occurred in 7 patients out 23 who showed the long-term healing, after a mean period of 7.29 ± 3.45 months. The prognostic score (University of Connecticut Osteonecrosis Numerical Scale—UCONNS) was significantly higher (*p* = 0.01) in patients with poor healing as compared to complete healing, both at 1 and 3 months posttreatment. Conclusions: A MRONJ treatment approach based on a combined pharmacological and surgical treatment strategy showed a high rate of healing and few recurrences.

## 1. Introduction

As defined in 2014 by the American Association of Oral and Maxillofacial Surgeons (AAOMS), medication-related osteonecrosis of the jaw (MRONJ) is a severe side effect of bisphosphonates and of certain anti-resorptive drugs, such as denosumab [1], commonly prescribed for controlling severe osteoporosis, Paget’s disease, and bone malignancies, including multiple myeloma and bone metastases [1]. Bisphosphonates (BPs) are analogues of inorganic pyrophosphate, inhibiting the pyrophosphate-dependent enzymes mediating bone resorption [2]. Denosumab is a human monoclonal immunoglobulin G2 subclass (IgG2) that mimics the function of the endogenous molecule osteoprotegerin (OPG), reducing bone metabolism [3]. Even if the potential of BPs and denosumab to increase the survival in cancer patients remains uncertain, they significantly improve the quality of life, reducing bone pain in cases of advanced bone metastases [4].

MRONJ pathogenesis is still largely unknown. A multi-factorial mechanism has been advocated, involving inhibition of angiogenesis and remodeling in bone tissue, presence of continuous micro-trauma within the oral cavity during eating and speaking, as well as a potential role and impact from oral mucosal inflammation or odontogenic infection [5,6].

A recognized risk factor for MRONJ is high concentration and long duration of BP intake [7]. The frequency of MRONJ in cancer patients has been estimated, ranging from 1% to 15%, while the frequency in patients with osteoporosis, receiving much lower BP doses, is estimated around 0.001% to 0.01% [8,9]. Antiangiogenic agents, tyrosine kinase inhibitors, and monoclonal antibody-targeting Vascular-Endothelial Growth Factor (VEGF)—such as sunitimib, sorafefenib, bevacizumab—worsen the risk of MRONJ from 5- to 10-fold [1,10]. Since MRONJ negatively impacts patient quality of life [11], preventive dental treatment is strongly recommended [12].

Despite no international consensus for treating MRONJ, a decisional tree to manage these patients requires consideration of the staging of MRONJ, patient age, gender, and systemic health [9]. The primary objective of treatment is to control symptoms, mainly pain, and to avoid progression of MRONJ to a more advanced stage [13]. Recent studies suggest a need for early surgical management to ensure complete removal of the necrotic bone following implementation of a first-line conservative nonsurgical approach with antibiotics, antimicrobials, and analgesics [13,14,15,16,17]. Surgery, in particular, is recommended in the presence of well-defined bone sequestra; in these cases, sequestrectomy or surgical *debridement* is needed. In patients with advanced stages who show a progression of the disease, or in cases of persistent pain and infection despite the medical therapy, an extensive resection is required [1,18,19]. Medical therapy itself appears to control pain and infection in about 50% of patients. A risk of sepsis, mainly in immunocompromised cancer patients, further justifies surgical intervention [19]. To date, MRONJ surgical therapy has been associated with variable percentages of success due to high heterogeneity among published studies [18,20,21].

The aim of this study was to retrospectively assess the success and recurrence rates in a cohort of MRONJ patients treated with a first pharmacological phase, intended for isolating gradually necrotic bone tissue and promoting sequestration, followed by a surgical intervention limiting the need for extensive resections.

## 2. Materials and Methods

### 2.1. Patients

#### 2.1.1. Study Design and Patient Population

This cohort study retrospectively analyzed clinical records of MRONJ patients referred to the Oral Medicine Unit (ASST Santi Paolo e Carlo) at University of Milan, from October 2008 to December 2017. MRONJ staging of affected patients was defined according to 2014 AAOMS criteria [1].

#### 2.1.2. Eligibility Criteria

The inclusion criteria included patients with MRONJ diagnosis at stage I–III, according to AAOMS criteria [1,21]. All patients were treated first with a pharmacological phase and then a surgical phase for bone sequestration removal [1,13,14,15,16,17]. The exclusion criteria were [1]: history of radiation therapy to the jaws or obvious metastatic disease to the jaws, no history of pharmacological therapy for MRONJ, and no history of surgical removal or spontaneous exfoliation of bone sequestra.

#### 2.1.3. Treatment Intervention

Each patient received two phases of management, i.e., a pharmacological phase and a surgical phase. Based on previous literature [1,13,14,15,16,17], the protocol first used a medical management approach with antibiotics and local measures and followed the patients until there was evidence of bone sequester formation. At that point, surgical treatment of the MRONJ lesion site was performed with the goal of removing the sequestered bone and debridement of the site. In recurrent cases, the patients were referred to maxillofacial surgeons for major surgical procedures, i.e., bone resection. 

Based on previous studies [1,9,21,22], systemic antibiotics were prescribed to all study patients as follows: amoxicillin 3 g/day or clindamycin 1800 mg/day in cases of allergy to the penicillin; for the cases scarcely responsive to single-antibiotic therapy, metronidazole 500 mg/day for a maximum 14 days. Topical antiseptic therapy with 0.2% chlorhexidine mouthwash and 1% chlorhexidine gel, applied onto exposed necrotic bone, was also prescribed [17].

Surgical intervention (sequestrectomy) was performed when sequestered bone was clinically or radiographically evident and not spontaneously exfoliated, following a MRONJ protocol previously recommended [23]. Briefly, one week before surgical intervention, each patient received the dental scaling, topical antiseptic therapy (0.2% chlorhexidine mouthwash, twice/day), and the prescription of antibiotic therapy started three days before the surgery (amoxicillin 3 g/day, or clindamycin 1800 mg/day in case of allergy to penicillin) [23]. On the day of surgical intervention, under local anesthesia, necrotic bone was removed via full-thickness mucoperiosteal flap, with minimal trauma to the cortical plates. Teeth involved in the necrotic area were extracted and a meticulous bone curettage and osteoplasty were performed until clear bleeding and white vital bone were clinically evident. The flap was closed with an absorbable suture via periosteal releasing incisions to achieve primary closure and in order to maximize the vascular supply to the area as well as to reduce risk of infection at the surgical site. Post-surgery, patients continued for two weeks the systemic antibiotic therapy and antiseptic mouthwash and also applied 1% chlorhexidine gel onto the surgical wound twice/day for at least 14 days. On the basis of the promising results obtained in previous studies [24,25,26], and under approval of the patient’s oncologist, pentoxifylline and tocopherol were also prescribed per os (pentoxifylline 800 mg/day + tocopherol 800 U.I./day), before and/or after surgical intervention, according to clinical case.

### 2.2. Data Collection 

Clinical and demographic data collected for each patient included, age, gender, systemic conditions, MRONJ stage [1], bisphosphonate, anti-resorptive, or anti-angiogenetic therapy (dosage, suspension, and duration), formation of bone sequestra, area of exposed bone (localization and size), date of surgical intervention(s), number of recurrences, and length of follow-up. The duration of therapy was determined as the period from the start of treatment to the first visit to our clinical unit. The prognostic score (University of Connecticut Osteonecrosis Numerical Scale—UCONNS) described by Landesberg was applied to find possible correlation between outcomes and patient systemic conditions [27]. UCONNS scores assess the individual prognosis based on known risk factors for MRONJ management failure, including systemic health conditions, comorbidities, type, and duration of bisphosphonate therapy and type of intervention performed. UCONNS scores were categorized as follow: 0–8, 9–16, 17–24, 25–32.

### 2.3. Outcomes

#### 2.3.1. Primary Outcomes: Clinical Healing 

The following criteria for clinical healing were used (adapted from [28]):

- Short-term healing—A patient was defined as “healed at short-term”, if presenting, for at least 1 month after sequestrectomy or spontaneous exfoliation of necrotic bone, the following clinical picture: absence of exposed necrotic bone or bone that can be probed through a fistula; absence of purulent drainage; absence of edema and stimulated pain; complete mucosal coverage of the surgical site.
- Long-term healing—A patient was defined as “healed at long-term”, if presenting the same clinical picture described above but lasting for at least 3 months after sequestrectomy or spontaneous exfoliation of bone sequestration.
- Stable MRONJ clinical picture—A patient was “stable” when, at the last available follow-up visit, showing clinical evidence of MRONJ, with the same stage seen during the first visit.
- Worsened MRONJ clinical picture—A patient was “worsened” when, at the last available follow-up visit, showing clinical evidence of MRONJ, with a worse stage than found at first diagnosis.
- Improved MRONJ clinical picture—A patient was “improved” when, at the last available follow-up visit, showing clinical evidence of MRONJ, with a better stage than the one of the first diagnosis.

#### 2.3.2. Secondary Outcomes: Rate of MRONJ Recurrence

##### Recurrence

Recurrence was defined as the appearance of exposed necrotic bone or bone that could be probed through a fistula, in association or not with radiographic evidence of architectural bone changes persisting for more than 8 weeks in an area that had already demonstrated a long-term healing.

##### Adverse Effects

Any adverse events due to pharmacological and/or surgical phases were recorded when specified in the medical record.

### 2.4. Statistical Analysis

Means and standard deviations were calculated for continuous variables; Kolmogorov–Smirnov test of normality was applied, and data were normally distributed. A *t*-test was used to compare means between two unpaired samples. For categorical variables, extracted data were expressed as percentages, and statistical analyses to identify significant differences were performed by applying the χ^2^ test using the online Graphpad statistical software (GraphPad Software^®^, San Diego, CA, USA). Statistical significance was set at *p* ≤ 0.05. Odds ratios were also calculated using the online MedCalc Software Ltd statistical software (MedCalc Software Ltd., Ostend, Belgium). 

### 2.5. Ethical Approval 

The study was performed under ethical approval obtained from the Ethic Committee of the AO San Paolo (ID study approval: ONM-BF-Gene, 2016). Opt-out patient consent was obtained.

### 2.6. STROBE Statement

The Strengthening the Reporting of Observational studies in Epidemiology (STROBE) statement was used to prepare this report.

## 3. Results

**Figure 1:**
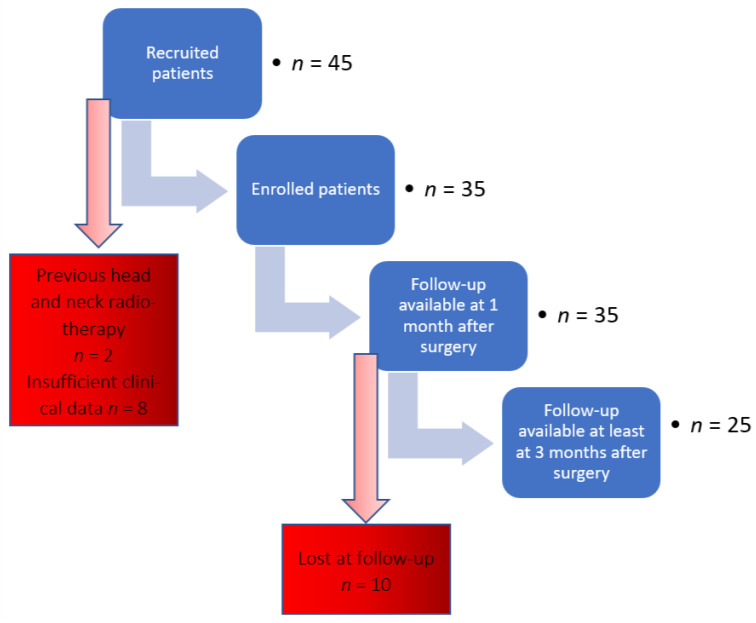
Flow diagram of enrolled patients.

From an initial cohort of 45 patients with MRONJ, 35 subjects were included in the study. Ten patients were excluded for the following reasons: positive anamnesis for head and neck radiotherapy (*n* = 2), or insufficient clinical data (*n* = 8). Figure 1 provides a flow-chart of enrolled study patients, while patient demographic and clinical data are summarized in Table 1. 

Most of patients were women (χ^2^; *p* = 0.02); the mean age of study participants at the first examination was 73.46 ± 9.29 years (range 51–93 years) (Table 1). Six patients out thirty-five were undergoing anticancer chemotherapy at the moment of surgical intervention. Eight patients were receiving intravenous steroid treatment, which in four cases was associated with the chemotherapeutic regimen (Table 1). Six patients were also affected by diabetes. 

Most patients were treated with zoledronate and showed stage II MRONJ lesions (Table 1). 

The mean follow-up of patients, from the first visit up to the last one available, was 23.86 ± 18.14 months (range: 1–74 months). The mean therapy with zoledronate lasted 34.29 ± 33.42 months; in some cases, the drug was suspended for a mean period of 8.53 ± 20.21 months. The mean duration of alendronate therapy was longer (79.42 ± 63.33 months), with a mean suspension period, when occurring, of 13.15 ± 19.58 months. The mean duration of the therapy with denosumab was shorter, lasting 15 ± 7.94 months, with a mean suspension time of 0.8 ± 1.1 months (Table 2). 

The pharmacological phase involved the use of topical chlorhexidine and systemic amoxicillin, with 11 patients also receiving metronidazole. In nine patients (25.71%), the supportive pharmacological therapy for MRONJ included also pentoxifylline and tocopherol (mean period of administration 3.81 ± 2.46 months). 

Seven cases showed spontaneous exfoliation of necrotic bone. In four cases, the exfoliation occurred during the pharmacological phase: in one case, the sequestrum was completely exfoliated not requiring any further intervention, while in three patients the sequestra were partially exfoliated, requiring the further surgical intervention at the same site. In the other three patients, a spontaneous exfoliation of an additional bone sequestrum followed sequestrectomy. 

Fifty-seven interventions of sequestrectomy were performed. Eighteen patients received a single surgical intervention, while four patients underwent two interventions in two different sites affected by MRONJ. In five subjects, the interventions were more than two; one patient required five interventions at the same site. 

**Figure 2:**
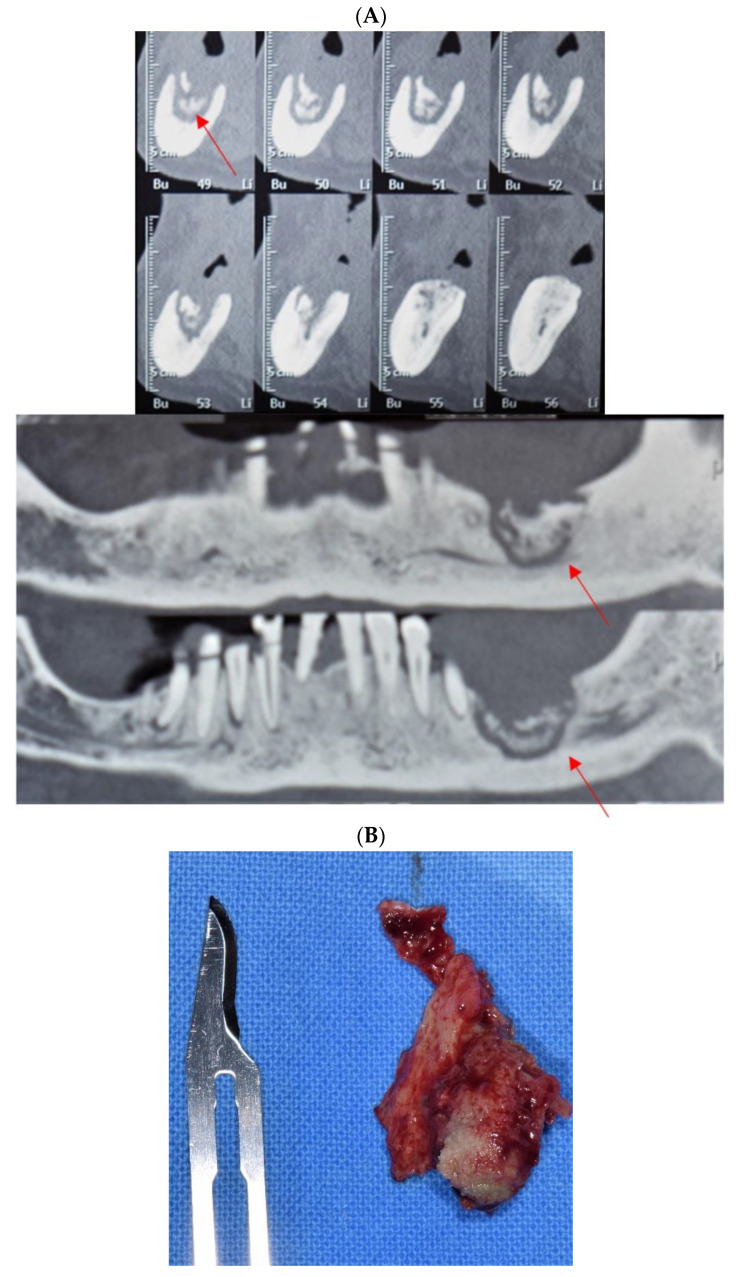
Clinical case of MRONJ localized in left mandible. (**A**) Cone beam computed tomography (CBCT) images, sagittal and frontal views, showing isolation of mandibular bone sequestration following antibiotic and topical antiseptic therapy (red arrows indicate the bone sequestrum). (**B**) Necrotic bone sequestration, resulting from the surgical intervention.

Figure 2 describes a clinical case where antibiotic administration gradually resulted in the isolation of bone sequestrum from the surrounding healthy bone. 

**Figure 3:**
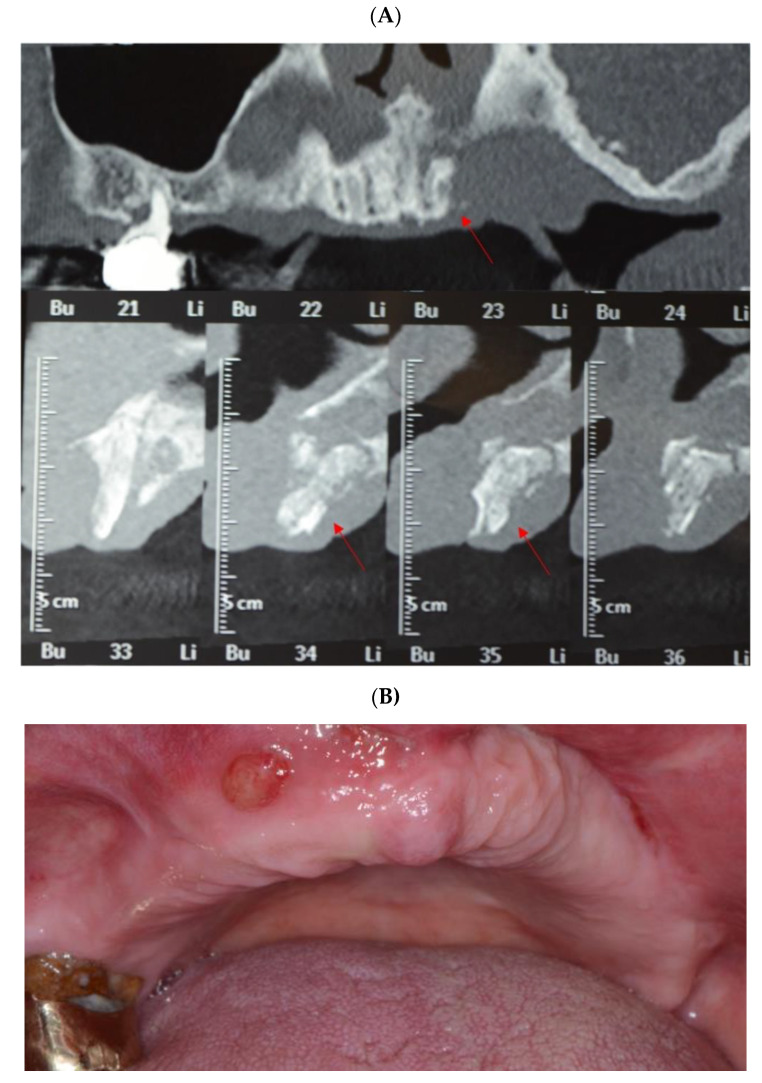

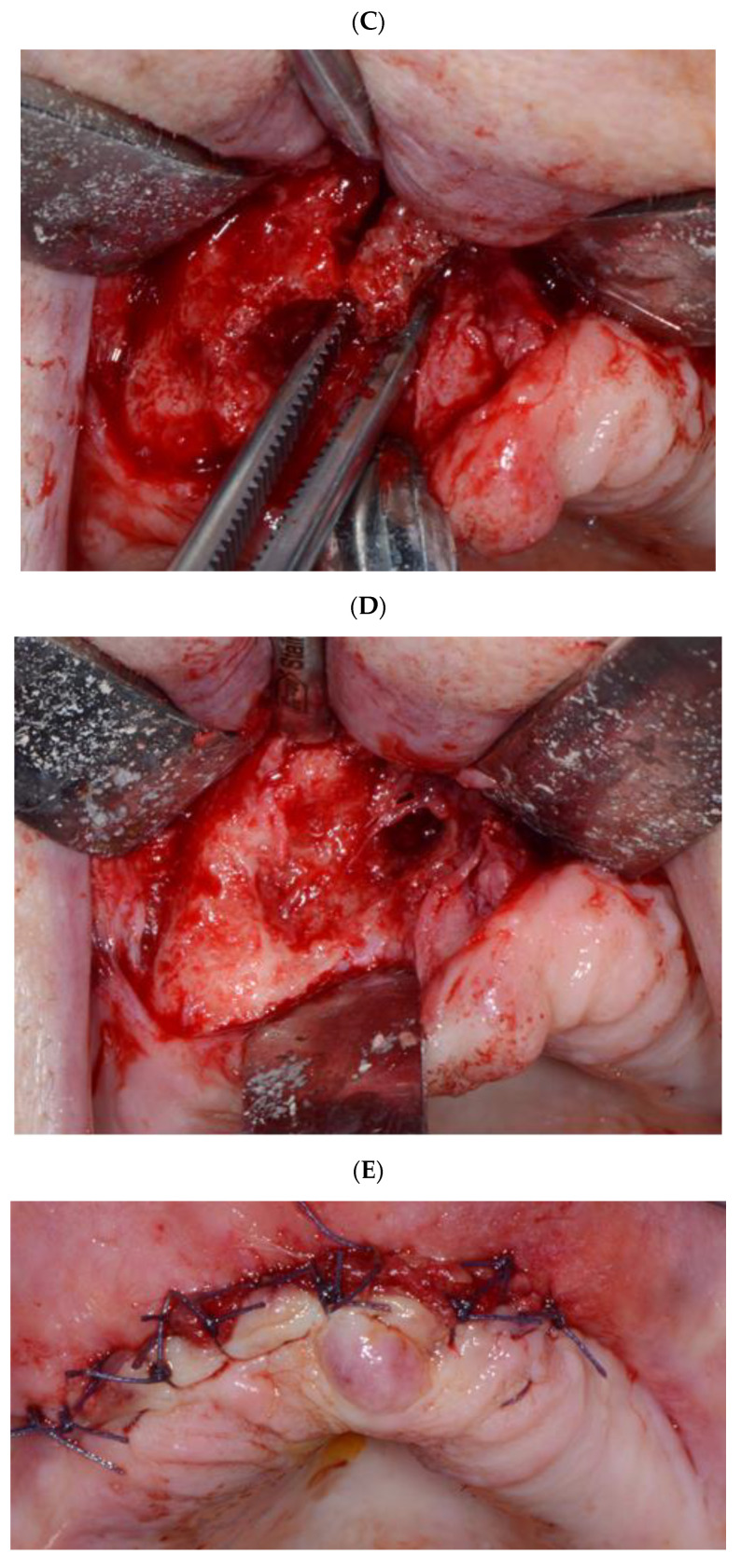

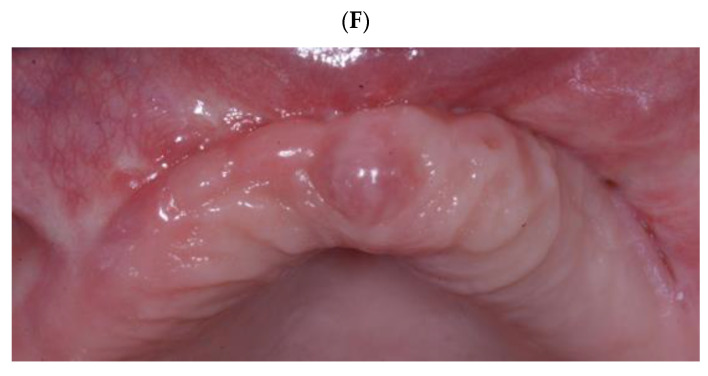
Clinical case of MRONJ localized in the upper maxilla. (**A**) Cone beam computed tomography (CBCT) images, frontal and sagittal views, showing maxillary bone sequestration (red arrows indicate the bone sequestrum). (**B**) Intraoral clinical view showing the presence of fistula, which demonstrates the presence of infection. (**C**) Necrotic bone sequestrum removal, after the opening of the surgical flap (mid-crestal incision on the alveolar crest of the edentulous area). (**D**) The necrotic bone was completely removed until reaching the healthy bone tissue; bone curettage and osteoplasty were performed until vital bone was clinically observed. (**E**) Primary closure via periosteal releasing incisions using absorbable suture. (**F**) Follow-up after 3 months from the surgical intervention showing a complete tissue healing.

Figure 3A–F describes the surgical intervention for bone sequestrum removal in a patient who developed MRONJ after zolendronate intake for oncological reasons (metastases of breast carcinoma).

### 3.1. Primary Outcomes

#### 3.1.1. Short-Term Healing

**Figure 4:**
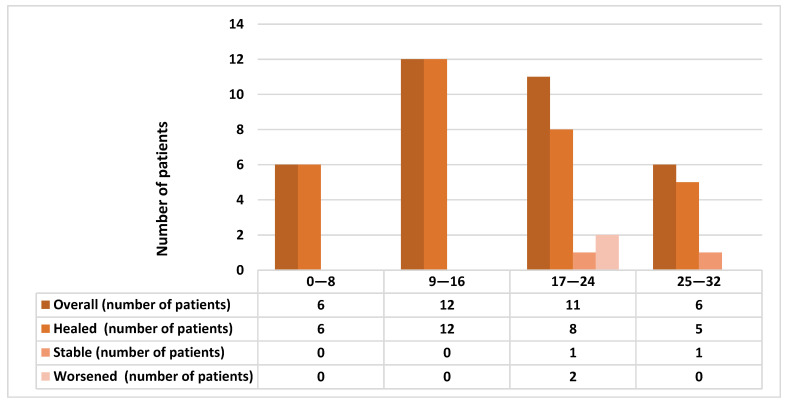
Comparison of the short-term healing outcomes depending on University of Connecticut Osteonecrosis Numerical Scale (UCONNS) score.

At 1-month post-surgery, 31 out 35 (88.57%) patients showed complete healing. The four not-healed cases were MRONJ stage II: in two cases the picture was stable at last follow-up, while the other two demonstrated a worsening of their conditions. These four patients received zolendronate (in one case alternating to denosumab) for oncological reasons for a mean period of 18.66 ± 4.72 months (range 17–60 months). Two of them had suspended the drug for 10 and 13 months. The mean UCONNS prognostic score was 15.83 ± 7.24 in healed patients, 25 ± 4.08 in not-healed patients (*t*-test, *p* = 0.01). Figure 4 describes the short-term healing outcomes depending on UCONNS score.

#### 3.1.2. Long-Term Healing

**Figure 5:**
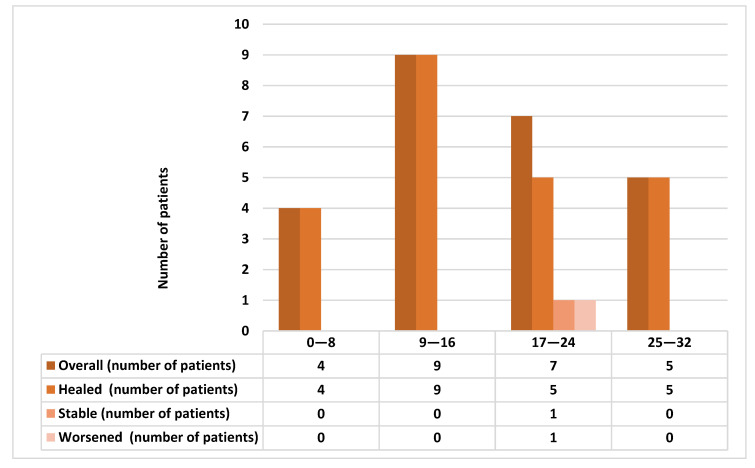
Comparison of the long-term healing outcomes depending on UCONNS score.

Twenty-five patients had long-term follow-up (at least 3 months), while the other ten patients, including two patients who did not heal at short-term, were lost prior to further follow-up. In the 25 patients, the mean follow-up was 27.28 ± 15.37 months from the first visit. Twenty-three out 25 (92%) showed complete healing of the surgical site for at least 3 months after surgery (Figure 5). Two patients were not healed at either short-term or long-term follow-up. One of them showed a stable MRONJ lesion in the posterior maxilla, while the other patient with MRONJ at the mandible showed a worsening clinical picture. This patient was referred to maxillofacial surgeons for major surgery. Both not-healed patients had MRONJ stage II and received zoledronate for oncological reasons, respectively for 24 and 17 months. One of the patients was under current zoledronate treatment (in addition to chemotherapy), while the other patient had suspended the drug 13 months before. The mean UCONNS prognostic score was 16.56 ± 7.63 in healed patients, as compared to 22.5 ± 0.7 in not-healed patients (*t*-test, *p* = 0.001).

Table 3 reports healing outcomes at long term in patients who received also pentoxifylline and tocopherol therapy.

### 3.2. Secondary Outcomes

#### 3.2.1. Recurrences

**Figure 6:**
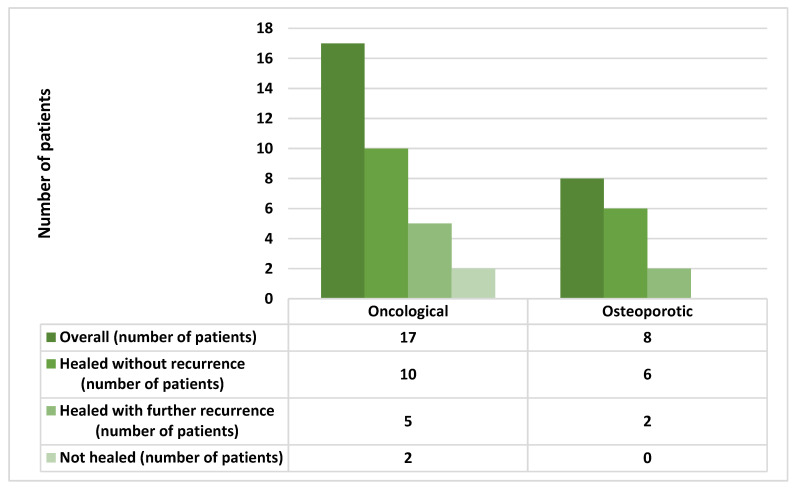
Comparison of long-term healing and recurrence in oncological and osteoporotic group.

Recurrences were recorded in seven patients out 23 (30.4%) who showed long-term healing: two were stage I, four at stage II, and one was at stage III. The recurrences occurred on average 7.29 ± 3.45 months after surgical intervention. Five out of seven patients who showed recurrences were receiving zoledronate for a mean period of 37.2 ± 24.47 months, while the remaining two patients were under therapy with alendronate for a mean period of 84 ± 16.97 months (Odds Ratio—OR:1.81; 95% CI: 0.27 to 11.86; *p* = 0.53). Recurrence in the oncological group occurred in five out 15 healed patients, while considering the osteoporotic group in 2 out 8 healed patients (OR 1. 50; 95% CI: 0.21 to 10.30; *p* = 0.68) (Figure 6).

#### 3.2.2. Adverse Events

No adverse events were reported, except for one case of spontaneous bleeding from the exposed necrotic bone, which occurred soon after the start of pentoxifylline and tocopherol therapy.

## 4. Discussion

MRONJ is a debilitating condition that more frequently affects females, the elderly, and persons treated for oncological reasons [18,20,28]. Although this complication has important clinical implications for dental practitioners—who need to know the correct management of a patient under anti-resorptive therapy—recent studies highlighted a lack of knowledge among dentists and dental students [29,30,31].

These findings support that, following a combined pharmacological and surgical conservative approach, most patients experience a complete healing. The UCONNS prognostic score was significantly higher in patients with poor healing as compared to those with complete healing in lesions. This conservative approach of MRONJ management was based on previous study outcomes [1,9,21,22] and included systemic antibiotics as well as antiseptic therapy with 0.2% chlorhexidine mouthwash and 1% chlorhexidine gel applied onto exposed necrotic bone [17].

Treatment of MRONJ patients remains challenging, and therapeutic options vary from pharmacological supportive approach with antibiotics and antiseptics to extensive surgical resection of necrotic bone. According to previous studies [14,18,20,32,33], an early surgical approach with appropriate resection margins and primary wound closure can ensure a better surgical outcome (mucosal healing without signs of infection) stage improvement at 6 months, with mucosal and radiographic healing evident at one-year posttreatment. Since pharmacological therapy alone rarely leads to lesion healing even at stage I [16], Khan and colleagues [9], in their systematic review, recommended surgical resection with tension-free primary closure.

In our experience, pharmacological management of MRONJ lesions seems to promote progressive isolation of the bone sequestrum, enabling minimally invasive surgical intervention, with a potentially higher rate of long-term success than major surgical resection. Progressive isolation of necrotic bone throughout the preliminary pharmacologic phase allows removal of necrotic tissue without undue sacrifice of healthy bone.

Consistent with previous research findings on conservative management of MRONJ [16,17,21], a high healing rate was achieved in the present study, although the study sample size was small. Importantly, UCONNS scores were confirmed to play a significant role in influencing treatment outcomes. Indeed, similar to a proposed cut-off value of UCONNS scores ≥ 15 to identify a higher rate of therapeutic failure [34], we found that all non-healed (both stable and worse) patients had a UCONNS score beyond 17.

The rate of recurrence after 3-month healing (30.4%), in the present study, appeared similar to that reported by Mucke and colleagues (28.7%), although they performed only surgical debridement in most of their patients [32]. However, due to methodological heterogeneity, a direct comparison among studies remains still complicated, considering that recurrence may occur after several months and that a too short follow-up period may bias the findings.

The additional use of pentoxifylline and tocopherol in treatment of MRONJ lesions is worth further investigation. In this study, most patients healed without major adverse side effects. Only one patient experienced bleeding at the start of pentoxifylline and tocopherol therapy. The Italian drug agency (AIFA) [35] warned of excessive bleeding among patients receiving anticoagulants, thrombolytic agents, and inhibitors of platelet aggregation who simultaneously are administered pentoxifylline and tocopherol. In our study the patient who developed spontaneous bleeding was not under treatment with any of these drugs.

The main limitation of this retrospective study is the use of medical records not specifically designed for the aim of the study. Thus, collected data might be limited in scope, making identification of potential confounding factors difficult, and making patient inclusion into the study prone to selection bias. Further limitations include varying lengths of follow-up of patients and varying patient compliance with the pharmacological protocol, along with the lack of calibration among the oral surgeons who performed the interventions and a lack of standardization in data collection. The retrospective design also predisposes the study to numerous threats to external validity, which limits interpretation and generalizability of the results (such as single-group threat, i.e., the lack of a comparison and/or control groups, and historical threat, where other events, different from the intervention under investigation, can affect the outcomes) [36].

## 5. Conclusions

An initial pharmacological phase based on antibiotic and antiseptic agents is useful to gradually isolate bone sequestration in MRONJ patients and facilitate a subsequent surgical phase. This approach is particularly advisable for treatment of stages I and II of MRONJ. UCONNS-related prognostic factors play a relevant role in determining the success of MRONJ therapy.

## Figures and Tables

**Table 1:** Demographic and clinical data concerning patient gender, age, comorbidities, concomitant cancer therapies, stage, and localization of medication-related osteonecrosis of the jaw (MRONJ) (*n* = 35).

Demographic and Clinical Data	Number of Patients (%)
**Gender**	
-Male	11 (31.4%)
-Female	24 (68.6%) *
**Age, years**	**Years**
Range	51–93
Mean, SD	73.46 ± 9.29
**Concomitant cancer therapies**	
-Steroids	4 (11.4%)
-Chemotherapy	6 (17.1%)
-Steroids and Chemotherapy	4 (11.4%)
-No steroids, No Chemotherapy	21 (60%)
**Primary disease requiring anti-resorptive drugs**	
-Breast cancer	10 (28.6%)
-Prostate cancer	4 (11.4%)
-Multiple myeloma	7 (20%)
-Osteoporosis	14 (40%)
**Type of drug associated with MRONJ**	
Zolendronate	17 (48.5%) *
Alendronate	9 (25.7%)
Denosumab	2 (5.7%)
Alendronate + Denosumab	2 (5.7%)
Alendronate + Risendronate	1(2.9%)
Alendronate + Zolendronate	1(2.9%)
Alendronate + Ibandronate	1(2.9%)
Ibandronate + Clodronate	1 (2.9%)
Zolendronate + Denosumab	1 (2.9%)
**Stage of MRONJ**	
-Stage I	6 (17.1%)
-Stage II	28 (80%)
-Stage III	1 (2.9%) *
**MRONJ localization**	
Maxilla	12 (34.2%)
Mandible	24 (68.5%) * ^ψ^

* χ^2^ test, significance: *p* ≤ 0.05. ^ψ^ One patient had both mandibular and maxillary lesions.

**Table 2:** Clinical data concerning MRONJ-related therapy.

MRONJ-Related Therapy	Months
**Duration of therapy**	
-Zoledronate	34.29 ± 33.42
-Alendronate	79.42 ± 63.33
-Denosumab	15 ± 7.94
**Suspension of drug**	
-Zoledronate	8.53 ± 20.21
-Alendronate	13.15 ± 19.58
-Denosumab	0.8 ± 1.1

**Table 3:** Clinical outcomes of patients who received pentoxifylline and tocopherol therapy for management of MRONJ.

Age (Years)	Cause of Anti-Resorptive Treatment	Gender	Type of MRONJ-Related Drug	UCONNS Score	Stage of MRONJ	Site of MRONJ	Outcomes
68	Cancer	Female	Alendronate	12	Stage II	Mandible	Healed
65	Cancer	Female	Zoledronate	23	Stage II	Mandible	Worsened
53	Cancer	Female	Zoledronate	31	Stage II	Mandible	Stable
51	Cancer	Female	Zoledronate-Denosumab	24	Stage II	Maxilla	Worsened
93	Osteoporosis	Female	Alendronate-Denosumab	13	Stage II	Maxilla/Mandible	Healed
85	Cancer	Female	Zoledronate	26	Stage II	Maxilla	Healed
65	Cancer	Male	Denosumab	22	Stage II	Mandible	Healed
90	Osteoporosis	Female	Alendronate	8	Stage II	Maxilla	Healed
77	Cancer	Male	Zoledronate	21	Stage II	Mandible	Healed

## Data Availability

The data presented in this study are available on request from the corresponding author.
